# Smile Attractiveness and Treatment Needs of Maxillary Midline Diastema with Various Widths: Perception among Laypersons, Dental Students, and Dentists in Malaysia

**DOI:** 10.1155/2023/9977868

**Published:** 2023-04-15

**Authors:** Nur Amirah Binti Mohamad Sabri, Sarah Batrisyia Binti Ridzwan, Suet Yeo Soo, Lishen Wong, In Meei Tew

**Affiliations:** Department of Restorative Dentistry, Faculty of Dentistry, The National University of Malaysia, Jalan Raja Muda Abdul Aziz, Kuala Lumpur 50300, Malaysia

## Abstract

Smile attractiveness and the need for treatment of maxillary midline diastema with various widths are perceived differently between dentally trained and nondentally trained individuals of different sociodemographic backgrounds. This study aims to evaluate how laypersons, dental students, and dentists in Malaysia differ in their perceptions on smile attractiveness and treatment needs of maxillary midline diastema. A smiling photograph with well-aligned maxillary central incisors with proportionate width-to-height ratio and healthy gingival tissues was selected and digitally manipulated to create maxillary midline diastema with 0.5, 2.0, and 4.0 mm widths. The smile attractiveness and the perceived need for treatment of varying widths of maxillary midline diastemas were rated by laypersons, dental students, and dentists using the Likert scale via a single set of self-administered questionnaires. The impact of sociodemographic variables on aesthetic perception of different gap widths was tested using univariate analysis followed by a multiple linear regression model. A total of 158 laypersons, 118 dental students, and 138 dentists participated in this study. Both laypersons and dentists showed significantly higher mean aesthetic scores for 0.5 mm maxillary midline diastema, lower mean aesthetic scores, and hence higher mean treatment needs scores for 4.0 mm maxillary midline diastema as compared with dental students (*p* < 0.05). In general, female respondents perceived a gap width of up to 2.0 mm as aesthetically pleasing. Higher educational group and the Malay ethnicity had tolerance threshold of 0.5 mm gap width. The older group considered 4.0 mm gap width as aesthetically unpleasing. In conclusion, both laypersons and dentists accepted a 0.5 mm maxillary midline diastema as an attractive smile but considered 4.0 mm maxillary midline diastema as unpleasing smile which required treatment. Perceptions of laypersons and dentists were significantly different from dental students. Educational level, gender, ethnicity, and age were significantly associated with smile attractiveness of maxillary midline diastema at different investigated widths.

## 1. Introduction

Maxillary midline diastema (MMD) is clinically characterized as a gap or space between the two maxillary central incisors with prevalence ranges from 1.6% to 25.4% [1]. It is a common dental phenomenon that presents in growing children and requires no treatment [2]. However, MMD with different widths could persist until adulthood especially in clinical situations of high frenal attachment, presence of supernumerary teeth (mostly Mesiodens), teeth–jaw discrepancy, congenital missing lateral incisors, disrupted eruption of canine, and hereditary family history [3].

The perception of smile attractiveness varies among individuals with different backgrounds and experiences. For example, the smile attractiveness of MMD with various widths may be perceived differently by dental and nondental personnel from diverse sociodemographic backgrounds [4]. Additionally, the perception of smile discrepancy is highly subjective and can be influenced by cultural, ethnic, gender, and age factors among laypersons [1, 5]. However, experienced dental clinicians who have been well-trained to objectively analyze smile attractiveness can easily detect minor discrepancies in ideal smile parameters [6]. On the other hand, dental students may be less critical in identifying altered dental aesthetics due to their lack of clinical experience [7].

The aesthetic perception of MMD of varying widths differs in relation to age group, gender, level of education, and ethnicity [8]. A cross-sectional study by Umanah et al. [9] assessed the impact of sociodemographics on smile aesthetics and reported that the majority of Nigerian women, those with a younger age and tertiary level of education, perceived MMD as a beautiful smile characteristic and desired artificially created MMD. On the other hand, Enabulele and Ehis [10] showed that males had a higher preference for MMD than females. Among ethnic groups, the Jordanian population considered MMD unattractive [11] and required closure but Black West Africans with their unique characteristics and cultural norms [12] were in favor of MMD.

Different dental approaches have been suggested to treat various widths of MMD including composite build-up, veneers, crowns, and orthodontic treatment [13]. Of all the aforementioned treatment options, restoring MMD to an aesthetically pleasing result using direct composite build-up is challenging. To manage this challenging situation, Paolone et al. [14, 15] presented a novel clinical technique involving composite frame modification during layering procedures to enhance the aesthetic outcome of anterior composite restorations and fulfill patients' aesthetic expectations. The needs for treatment are mainly due to psychological and aesthetic reasons rather than functional reasons [16]. Laypersons commonly perceive the need to close the gap when MMD is more than 2 mm, which negatively impacts dental appearance [17]. From a dental clinician's perspective, the need to treat MMD generally involves striking a balance between patients' expectations and a normative need after objective assessment using dental aesthetic indices [18, 19].

In light of the growing demand for dental aesthetics among patients, it is crucial to have a comprehensive understanding of how dental and nondental personnel perceive dentofacial aesthetics. Therefore, this study aims to assess the different perceptions of laypersons, dental students, and dentists regarding the aesthetic scores of simulated MMD, the effect of sociodemographic variables on aesthetic scores of MMD with various gap widths, and the treatment needs perceived by the surveyed groups.

## 2. Materials and Methods

This was a cross-sectional study which recruited participants aged 18 years old and above who were willing to take part in this study. This study was carried out in the Dental Faculty, The National University of Malaysia, and 10 private dental clinics in Kuala Lumpur. All participants were divided into three different groups: group 1 (laypersons), group 2 (undergraduate dental students), and group 3 (dentists). For group 1, a list of patients who attended the undergraduate clinics in the Dental Faculty, The National University of Malaysia, and private dental clinics from January 2022 to June 2022 was retrieved from registration data. For group 2, a list of all year 3 to year 5 undergraduate dental students was obtained from Dean office, Dental Faculty of The National University of Malaysia. For group 3, all dentists who were working in the Dental Faculty, The National University of Malaysia, and private dental clinics were listed. Laypersons, dental students, and dentists with odd number on the list were then selected to participate in this study. Informed consents were obtained from all the participants prior to the start of the study. This study was approved by the Institutional Research Board committee of The National University of Malaysia.

**Figure 1:**
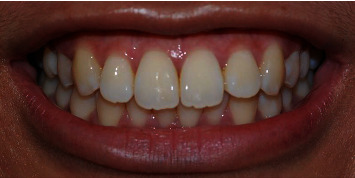
Photograph of smile characteristics close to standard norms.

**Figure 2:**
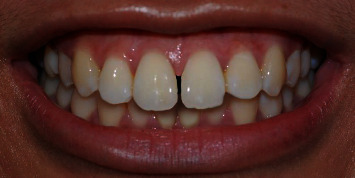
Photograph of digitally manipulated 0.5 mm maxillary midline diastema.

**Figure 3:**
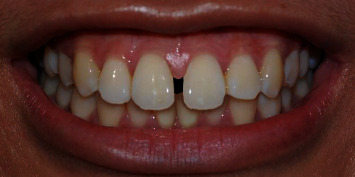
Photograph of digitally manipulated 2.0 mm maxillary midline diastema.

**Figure 4:**
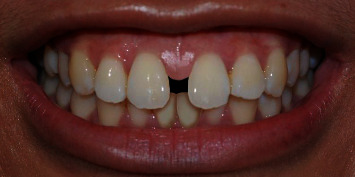
Photograph of digitally manipulated 4.0 mm maxillary midline diastema.

A photograph of smile characteristics close to standard norms [11] (Figure 1) was obtained using a digital single-lens reflex camera (Nikon D40, Japan Optical Industries Co., Ltd.) from a female undergraduate dental student from the Dental Faculty, The National University of Malaysia. Informed consent was obtained from her to digitally manipulate her smile photograph for rating purposes in this study. The original smile photograph was digitally manipulated using Adobe Photoshop software (Adobe Photoshop CS6) to generate MMD with widths of 0.5 (Figure 2), 2.0 (Figure 3), and 4.0 mm (Figure 4) without altering the crown length and width of maxillary central incisors, gingival display, buccal corridor width, smile arch, and upper-lip curvature.

### 2.1. Questionnaire

A questionnaire was prepared using an online survey Google form and distributed to selected participants. The questionnaire consisted of two parts: the first part included sociodemographic items such as age, gender, ethnicity, education level, history of having MMD, and category (laypersons, dental students, or dentists); the second part comprised a set of colored photographs showing digitally manipulated MMD with widths of 0.5, 2.0, and 4.0 mm.

Smile attractiveness of each manipulated photograph was rated by all participants using a rating scale (1 = aesthetically very unpleasing, 2 = aesthetically unpleasing, 3 = acceptable, 4 = aesthetically pleasing, 5 = aesthetically very pleasing) [11]. Each participant was required to self-rate the degree of agreement on the need to treat MMD with widths of 0.5, 2.0, and 4.0 mm based on manipulated photographs using a rating scale (1 = strongly disagree, 2 = disagree, 3 = neutral, 4 = agree, 5 = strongly agree).

### 2.2. Reliability of the Questionnaire

Twenty participants were randomly selected and requested to complete the questionnaire again after a 2-week interval. The reliability of each question was measured using test–retest reliability test.

### 2.3. Statistical Analysis

All the collected data were analyzed using SPSS software (version 13.0). Descriptive analysis was conducted to describe the sociodemographic data of respondents according to category. Differences of mean aesthetic and treatment needs score of maxillary midline diastema between laypersons, dental students, and dentists were tested using one-way ANOVA with post hoc comparison analysis performed if needed. The level of statistical significance was set at 0.05.

A two-step approach was used to analyze sociodemographic variables that are highly associated with aesthetic perception of 0.5, 2.0, and 4.0 mm MMD. The included explanatory variables were the following: gender (male = 0; female = 1), age (20–29 = 0; 30–39 = 1; 40–49 = 2; 50–59 = 3), races (Malay = 0; Chinese = 1; Indian = 3; others = 4), educational level (secondary school = 0, foundation = 1, bachelor's degree = 2; master's degree and above = 3), and history of having maxillary midline diastema (yes = 0, no = 1). First, univariate analysis was used to test the relationship between mean aesthetic scores of 0.5, 2.0, and 4.0 mm maxillary midline diastema and the associated sociodemographic variables. The variables (*p* < 0.25) in the univariate analyses were entered into a multiple linear regression model in a backward fashion to further analyze the factors that strongly affect the aesthetic perception of various widths of maxillary midline diastema. The level of statistical significance was set at 0.05.

## 3. Results

A total of 414 participants (158 laypersons, 118 dental students, 138 dentists) with age ranges between 20 and 59 years old participated in this study. The distribution of gender, age, race, educational level, and history of having MMD based on category is shown in Table 1. Majority of the participants were Malays. Most had received education up to foundation and above and had no MMD.

Differences in mean aesthetic score of 0.5, 2.0, and 4.0 mm MMD between three categories have been presented in Table 2. A significantly different mean aesthetic score between laypersons, dental students, and dentists was found in 0.5 and 4.0 mm MMD (*p* < 0.05). For multiple group comparisons, both laypersons and dentists had significantly higher mean aesthetic scores for 0.5 mm MMD but lower mean aesthetic scores for 4.0 mm MMD as compared with dental students.

The relationship between mean aesthetic scores of 0.5, 2.0, and 4.0 mm MMD and sociodemographic variables has been demonstrated in Table 3. Univariate analysis showed different sociodemographic variables significantly affected the mean aesthetic score of 0.5 mm (gender, ethnicity, and educational level), 2.0 mm (gender), and 4.0 mm (age, educational level, and history of having MMD) MMD (*p* < 0.25) and this had been further determined from the results of multiple linear regression analysis in Table 4. A significantly higher mean aesthetic score of 0.5 mm MMD had been shown in female respondents by 0.20 (*p* < 0.05) and those who received higher education by 0.21 (*p* < 0.05). The mean aesthetic score was significantly lower for ethnicity with higher value by 0.12 (*p* < 0.05). Females had a significantly higher mean aesthetic score by 0.11 (*p* < 0.05) for 2.0 mm MMD. As for 4.0 mm MMD, those with older ages had a significantly lower mean aesthetic score by 0.10 (*p* < 0.05).

**Figure 5:**
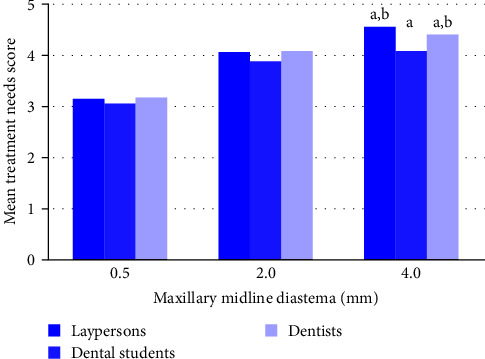
Treatment needs of maxillary midline diastema perceived by laypersons, dental students, and dentists. (a) Significant difference of mean treatment needs score between groups, *p* < 0.05. (b) No significant difference of mean treatment needs score between groups, *p* > 0.05.

Comparison of treatment needs of 0.5, 2.0, and 4.0 mm MMD between laypersons, dental students, and dentists has been illustrated in Figure 5. All three groups strongly agreed that 0.4 mm MMD needed to be corrected.

Intraclass correlation coefficient (ICC) of all measured variables showed good reliability with values ranging from 0.806 to 0.960.

## 4. Discussion

Dental and nondental individuals from different sociodemographic backgrounds may have a different aesthetic perception of MMD, and this can affect the perception of the need for MMD closures. Therefore, this study was conducted to assess the perception of laypersons, dental students, and dentists on smile attractiveness and treatment needs of digitally manipulated mild (0.5 mm), moderate (2.0 mm), and severe (4.0 mm) MMD [20]. Our study revealed that both laypersons and dentists had same perceptions of smile attractiveness of 0.5 and 4.0 mm MMD as well as the need to close 4.0 mm MMD. The perceptions of these two groups were significantly different than that of dental students. Sociodemographic factors such as educational level, gender, age, and ethnicity were significantly associated with aesthetic perception of MMD at the investigated widths.

In this study, smile attractiveness was perceived differently among laypersons, dental students, and dentists when the MMD increased from 0.5 to 4.0 mm. An MMD with a gap width of 0.5 mm is considered a minor dental irregularity and is generally accepted by laypersons and dentists in the study. Although both laypersons and dentists have different levels of dental knowledge exposure, they agree that an MMD <2.0 mm does not significantly impact overall smile attractiveness [21, 22]. On the other hand, orthodontists who are more critical in observing aesthetic deviation perceived that a 0.5 mm MMD affects a woman's dental appearance [23].

Smile attractiveness of a 4.0 mm MMD is generally rated as fair to poor by laypersons, dental students, and dentists. However, it is interesting to note that dental students are less critical when evaluating the smile attractiveness of 4.0 mm gap width compared with both laypersons and dentists. This contradicts a study by Alhammadi et al. [24], which shows that the absence of MMD was mostly favored by dental students. In this study, dental students with minimal clinical exposure in this study have lower sensitivity toward dental aesthetic, possibly due to a curriculum that emphasized dental health and function improvement over dental aesthetic [25] Therefore, Armalaite et al. [26] suggested that dental students in their clinical years should be taught to be aware of the different perception in dental attractiveness between laypersons and dental professionals in order to address patient's expectations and needs toward dental aesthetics. On the other hand, dentists with higher clinical experience have the capacity to recognize smile discrepancies, identify patients' aesthetic requirements, and formalize an effective treatment plan for patients [27].

The aesthetic perception of MMD in varying widths was further investigated when the participants were grouped based on sociodemographic variables. The effect of sociodemographics was more profound in 0.5 mm MMD as compared with a wider gap width. Among the surveyed ethnic groups, Malays accepted minimal gap width of 0.5 mm as a feature of smile attractiveness, the findings were in agreement to Chaves et al. [23], in which the respondents from Brazil perceived those with 0.5 mm MMD as one of the most attractive. This was contrary to Talic et al. [28] who reported Saudi Arabians had a low tolerance threshold in MMD and considered minimal gap width of 0.5 mm less attractive.

The level of education has an impact on the perception of smile aesthetics of 0.5 mm MMD. The study showed those with higher education levels were less sensitive to minor deviations in smile attractiveness. This finding was consistent with a study by Dindaroğlu et al. [29], which showed that the aesthetic scores of social and spontaneous smiles decreased with increasing education levels. One possible reason for this could be improved self-esteem in individuals with higher education levels, leading to higher self-acceptance [30], even in the presence of mild dental anomalies.

The effect of gender on the aesthetic perception of MMD remains controversial. This study demonstrated a significant gender-related difference in the aesthetic smile of 0.5 and 2.0 mm MMD, with females showing a higher tolerance level of acceptable deviation. These findings are consistent with those reported by Abu Alhaija et al. [11]. However, Bolas-Colvee et al. [19] reported that Spanish women were more critical than men and could only accept a gap width of <1.5 mm. On the other hand, Aldeeri et al. [31] investigated the smile aesthetic perception of orthodontists, dentists, and laypersons in Saudi Arabia and found that gender did not have a significant effect on perceived smile attractiveness.

Age is considered as another factor influencing smile aesthetics. The younger group in this study had a higher tolerance for accepting severe gap width of 4.0 mm compared with the older group. This is supported by a web-based study which reported that Caucasian respondents under 40 years old had a strong preference for a smile with an MMD [32] An increasing acceptance of MMD in youngsters could possibly be due to media influence. Lewis et al. [33] revealed that frequency of MMD appearing in photographs of Caucasian females in fashion magazines had been increasing, which in turn led to a change in readers' perception of beauty.

Closure of MMD could be carried out using orthodontic or/and restorative means. The decision to treat MMD is not solely dependent on the opinions of dental and nondental personnel, but also on the size of the gap. All investigated groups in this study agreed to treat MMD when the gap widths increased. However, for intergroup comparison, both laypersons and dentists showed higher agreement to close gap width of 4.0 mm than dental students. This is likely due to differences in aesthetic perception. Both laypersons and dentists considered a 4.0 mm gap width unattractive in a smile while dental students were more accepting.

There are several limitations in this study. Although photo digital manipulation is the most commonly used method to evaluate smile aesthetic perceptions, these simulated two-dimensional images are less ideal to entirely demonstrate the effect of maxillary midline diastema on dentofacial aesthetics compared with video presentation, which provides a more dynamic view of the face and smile. Apart from understanding the aesthetic perception of dental students and general dental practitioners, it will be interesting to further identify the tolerance threshold of dental specialists who commonly manage aesthetic cases like orthodontists, restorative specialists, and prosthodontists in future studies. To further evaluate aesthetic smile perception, it is important to determine other factors that impact an attractive smile such as incisors and gingival exposure, relationship between dental and facial midline, tooth proportions and tooth shade.

## 5. Conclusion

In conclusion, both laypersons and dentists accepted 0.5 mm MMD as an attractive smile but considered 4.0 mm MMD as an unpleasing smile that required treatment. Perceptions of laypersons and dentists were significantly different from dental students. Educational level, gender, ethnicity, and age were significantly associated with smile attractiveness of MMD at different investigated widths.

## Figures and Tables

**Table 1:** Distribution of participants' sociodemographic data by category.

	Laypersons (*N* = 158), *n* (%)	Dental students (*N* = 118), *n* (%)	Dentist (*N* = 138), *n* (%)
Gender			
Male	43 (27.8)	22 (18.6)	33 (23.9)
Female	115 (72.8)	96 (81.4)	105 (76.1)
Age (years)			
20–29	106 (67.1)	116 (98.3)	98 (71.0)
30–39	18 (11.4)	2 (1.7)	36 (26.1)
40–49	17 (10.8)	0 (0.0)	4 (2.9)
50–59	17 (10.8)	0 (0.0)	0 (0.0)
Ethnicity			
Malay	151 (95.6)	95 (80.5)	73 (52.9)
Chinese	3 (1.9)	16 (13.6)	45 (32.6)
Indian	1 (0.6)	6 (5.1)	18 (13.0)
Others	3 (1.9)	1 (0.8)	2 (1.4)
Educational level			
Secondary schools	12 (7.6)	0 (0.0)	0 (0.0)
Foundation	22 (13.9)	118 (50.8)	0 (0.0)
Bachelor's degree	102 (64.6)	0 (0.0)	122 (88.4)
Master's degree and above	22 (13.9)	0 (0.0)	16 (11.6)
History of having midline diastema			
Yes	2 (1.3)	11 (9.3)	14 (10.1)
No	156 (98.7)	107 (90.7)	124 (89.9)

**Table 2:** Mean aesthetic score of 0.5, 2.0, and 4.0 mm maxillary midline diastema (MMD) as perceived by laypersons, dental students, and dentists.

Midline diastema (mm)	Laypersons mean ± SD (*n* = 158)	Dental students mean ± SD (*n* = 118)	Dentists mean ± SD (*n* = 138)	*p*-value	Multiple comparison
0.5	2.47 ± 0.80	2.03 ± 0.70	2.27 ± 0.76	<0.001^*∗*^	LP, D > DS
2.0	1.61 ± 0.73	1.61 ± 1.03	1.52 ± 0.79	0.656	
4.0	1.20 ± 0.56	1.62 ± 1.20	1.33 ± 0.82	0.001^*∗*^	DS > LP, D

^*∗*^Significant difference of mean aesthetic score between laypersons (LP), dental students (DS), and dentists (D); *p* < 0.05.

**Table 3:** Relationship between different sociodemographic variables and mean aesthetic scores of 0.5, 2.0, and 4.0 mm maxillary midline diastema (MMD).

Variables	MMD 0.5 mm		MMD 2.0 mm		MMD 4.0 mm	
	Mean (SD)	*p*-value	Mean (SD)	*p*-value	Mean (SD)	*p*-value
Gender						
Male (0)	2.05 (0.75)	0.026^*∗*^	1.42 (0.76)	0.033^*∗*^	1.32 (0.78)	0.703
Female (1)	2.34 (0.76)		1.62 (0.83)		1.35 (0.82)	
Age (years)						
20–29 (0)	2.26 (0.77)	0.648	1.60 (0.85)	0.615	1.39 (0.87)	0.216^*∗*^
30–39 (1)	2.29 (0.85)		1.45 (0.66)		1.20 (0.55)	
40–49 (2)	2.38 (0.80)		1.52 (0.75)		1.24 (0.70)	
50–59 (3)	2.47 (0.72)		1.53 (0.72)		1.12 (0.33)	
Ethnicity						
Malay (0)	2.35 (0.78)	0.001^*∗*^	1.56 (0.75)	0.845	1.32 (0.74)	0.289
Chinese (1)	1.91 (0.66)		1.66 (1.09)		1.22 (0.14)	
Indian (2)	2.32 (0.80)		1.56 (0.92)		0.85 (0.17)	
Others (3)	2.17 (0.75)		1.50 (0.55)		0.00 (0.00)	
Educational level						
Secondary school (0)	2.00 (0.60)	<0.001^*∗*^	1.25 (0.45)	0.546	1.08 (0.29)	0.061^*∗*^
Foundation (1)	2.06 (0.75)		1.58 (0.92)		1.49 (0.96)	
Bachelor's degree (2)	2.39 (0.77)		1.59 (0.76)		1.29 (0.73)	
Master's degree and above (3)	2.45 (0.83)		1.53 (0.83)		1.24 (0.71)	
History of having midline diastema						
Yes (0)	1.57 (0.82)	0.850	1.56 (0.75)	0.905	1.59 (0.89)	0.140^*∗*^
No (1)	1.56 (0.75)		1.57 (0.82)		1.33 (0.80)	

Two simple *t*-test = gender, history of having midline diastema; one-way ANOVA = age, race, and educational level.  ^*∗*^Significant difference of mean attractive score between groups, *p* < 0.25.

**Table 4:** Factors associated with aesthetic score of 0.5, 2.0, and 4.0 mm maxillary midline diastema (MMD) (multiple linear regression model).

Factor(s)	*β* (SE)	Odds ratio (95% Cl)	*p*-value
Aesthetic perception 0.5 mm MMD^a^			
Educational level	0.24 (0.06)	0.21 (0.13–0.35)	<0.001^*∗*^
Gender	0.28 (0.09)	0.20 (0.10–0.50)	0.001^*∗*^
Ethnicity	−0.14 (0.06)	−0.12 (−0.26−0.32)	0.012^*∗*^
Constant	1.06 (0.28)		<0.001^*∗*^
Aesthetic perception 2.0 mm MMD^b^			
Gender	0.20 (0.09)	0.11 (0.02–0.39)	0.033^*∗*^
Constant	1.22 (0.17)		<0.001^*∗*^
Aesthetic perception 4.0 mm MMD^c^			
Age	−0.10 (0.05)	−0.10 (−0.20–0.01)	0.045^*∗*^
Constant	1.48 (0.81)		<0.001^*∗*^

^a^ *F* = 11.705; *df* = 410; *p* < 0.001; *R*^2^ = 0.08; adjusted *R*^2^ = 0.072. ^b^*F* = 6.985; *df* = 412; *p* = 0.033; *R*^2^ = 0.011; adjusted *R*^2^ = 0.009. ^c^*F* = 1.516; *df* = 411; *p* = 0.045; *R*^2^ = 0.009; adjusted *R*^2^ = 0.007.  ^*∗*^Significant difference of mean attractive score between groups, *p* < 0.05.

## Data Availability

The data used to support the findings of this study are available from the corresponding author upon request.

## Additional Points

*Clinical Significance*. An understanding of the perception of smile attractiveness and treatment needs of maxillary midline diastema between laypersons, dental students, and dentists of different sociodemographic backgrounds is of paramount importance for providing a comprehensive treatment plan.
